# Estrogen Receptor-α Mediates Diethylstilbestrol-Induced Feminization of the Seminal Vesicle in Male Mice

**DOI:** 10.1289/ehp.1103678

**Published:** 2012-01-24

**Authors:** Vickie R. Walker, Wendy N. Jefferson, John F. Couse, Kenneth S. Korach

**Affiliations:** 1 Receptor Biology Section, and; 2 Reproductive Medicine Group, Laboratory of Reproductive and Developmental Toxicology, National Institute of Environmental Health Sciences, National Institutes of Health, Department of Health and Human Services, Research Triangle Park, North Carolina, USA; 3 Taconic Inc., Germantown, New York, USA

**Keywords:** development, endocrine disruptor, reproductive tract

## Abstract

Background: Studies have shown that perinatal exposure to the synthetic estrogen diethylstilbestrol (DES) leads to feminization of the seminal vesicle (SV) in male mice, as illustrated by tissue hyperplasia, ectopic expression of the major estrogen-inducible uterine secretory protein lactoferrin (LF), and reduced expression of SV secretory protein IV (SVS IV).

Objectives: The present study was designed to evaluate the role of the estrogen receptor (ER) in this action by using ER-knockout (ERKO) mice.

Methods: Wild-type (WT), ERα-null (αERKO), and ERβ-null (βERKO) male mice were treated with either vehicle or DES (2 μg/day) on neonatal days 1–5. These mice were divided into two groups: In the first group, intact mice were sacrificed at 10 weeks of age; in the second group, mice were castrated at 10 weeks of age, allowed to recover for 10 days, treated with dihydrotestosterone (DHT) or placebo, and sacrificed 2 weeks later. Body weights and SV weights were recorded, and mRNA expression levels of *Ltf* (lactoferrin), *Svs4*, and androgen receptor (*Ar*) were assessed.

Results: In DES-treated intact mice, SV weights were reduced in WT and βERKO mice but not in αERKO mice. DES-treated WT and βERKO males, but not αERKO males, exhibited ectopic expression of LF in the SV. DES treatment resulted in decreased SVS IV protein and mRNA expression in WT males, but no effect was seen in αERKO mice. In addition, DES-treated βERKO mice exhibited reduced *Svs4* mRNA expression but maintained control levels of SVS IV protein. In DES-treated castrated mice, DHT implants restored SV weights to normal levels in αERKO mice but not in WT mice, suggesting full androgen responsiveness in αERKO mice.

Conclusions: These data suggest that DES-induced SV toxicity and feminization are primarily mediated by ERα; however, some aspects of androgen response may require the action of ERβ.

Studies in laboratory animals have unequivocally demonstrated that exposures to exogenous estrogens during certain periods of embryonic, fetal, and neonatal development lead to permanent and detrimental changes in reproductive function, most notably structural malformations among the reproductive tissues, reduced fertility, and cancer (Marselos and Tomatis 1992, 1993). Diethylstilbestrol (DES), a highly potent, orally available synthetic estrogen, is perhaps the most studied among the class of exogenous estrogens since being strongly associated with vaginal adenocarcinoma and abnormalities of the uterus and cervix in young women who were exposed via pharmacological use by their mothers during pregnancy (Herbst et al. 1971). Although DES was proscribed for use in women during pregnancy in 1973, these findings are the origin of the still currently held hypothesis that unintended exposures to compounds with estrogenic activities, such as phytoestrogens (e.g., genistein and coumestrol), pesticides [e.g., DDT (dichlorodiphenyltrichloroethane) and DDE (dichlorodiphenyldichloroethylene)], and plasticizers (e.g., bisphenol A), during embryonic through postnatal development may be linked to certain reproductive and other abnormalities in humans.

Many of the pathologies associated with developmental exposure to DES in humans can be replicated in outbred and inbred strains of laboratory mice (McLachlan et al. 1980; Newbold 2004). A few studies have focused on the health problems of men exposed to DES *in utero* (known as DES sons), and the results have been mixed. The most consistent finding indicates an increased risk for noncancerous epididymal cysts (Bibbo et al. 1977; Conley et al. 1983; Gill et al. 1979; Niculescu 1985; Wilcox et al. 1995); an increased risk of testicular cancer has not been ruled out but is not yet confirmed. The relevancy of the present protocol to DES sons is supported by previous studies in mice showing that exposure periods considered susceptible to perturbation by DES include exposure during gestation days (GDs) 9–16, on GD18 alone, and on postnatal days (PNDs) 1–5 (Newbold et al. 2000). Outbred female mice treated with DES as neonates develop a high incidence of uterine adenocarcinoma (Newbold et al. 1990); similarly treated male mice develop testicular cancer and abnormalities of the prostate and seminal vesicles (SVs) (McLachlan 1977). Since reports of these findings were published almost three decades ago, the fetal and neonatal mouse has been used extensively to investigate the toxicology of estrogenic compounds on reproductive tract development. Ostensibly, estrogen receptor-α (ERα) gene (*Esr1*)-knockout (αERKO) and ERβ (*Esr2*)-knockout (βERKO) mice have added to our ability to investigate the mechanisms by which exogenous estrogens exert their effects (Couse and Korach 1999). Conclusive studies have demonstrated that reproductive tracts of female αERKO mice are largely resistant to the developmental effects of neonatal exposure to DES (Couse et al. 2001). These data indicate a prominent role for ERα in mediating the toxicological effects of DES during reproductive tract development in females.

Reproductive tissues of male mice appear to be especially sensitive to the toxicological effects of DES (McLachlan et al. 1975; Prins 1992; Prins et al. 2001), and presumably other exogenous estrogens, throughout development. The development of the SV in the male reproductive tract involves three processes (Shima et al. 1990): growth, epithelial branching morphogenesis, and epithelial cytodifferentiation. In humans, SV development begins from the mesonephric (or Wolffian) duct at approximately 12 weeks of fetal age, and morphogenesis of the SV depends on fetal testicular androgens (Risbridger and Taylor 2006). At maturity, the SV consists of folded glandular epithelium with luminal secretory cells and a discontinuous layer of basal cells surrounded by a stromal layer of smooth muscle (Risbridger and Taylor 2006). In the mouse, the SV is present by embryonic day 16.5 (Lung and Cunha 1981) and develops from the mesonephric ducts. Morphogenesis begins on GD15, and during the first week of postnatal life the SV undergoes intense morphogenesis that results in the complex folded structures characteristic of the mature SV (Lung and Cunha 1981). ER is present in the mouse SV (Yamashita 2004) and appears during development on PND6 (Cooke et al. 1991). Significant increases in cytosolic ER levels in the SV have been reported in mice following neonatal exposure to DES (Turner et al. 1989).

In addition to morphological changes of the male reproductive tract, studies have shown that developmental exposure to estrogens leads to female-like patterns of gene expression in certain reproductive tissues during adulthood (Beckman et al. 1994; Newbold et al. 1989), including aberrantly high expression of progesterone receptor (*Pgr*), an estrogen-regulated gene, in the stromal cells of the male reproductive tract (Williams et al. 2000, 2001); ectopic expression of the major estrogen-inducible uterine secretory protein, lactoferrin (LF; also called lactotransferrin); and loss of constitutive expression of the androgen-regulated gene, SV secretory protein 4 (SVS IV), in the SVs (Beckman et al. 1994). To date, studies of DES exposure in ER-null male mice have focused largely on the prostate; these studies have demonstrated that ERKO males are refractory to the effects of neonatal DES exposure, whereas βERKO males exhibit susceptibilities comparable to similarly treated wild-type (WT) mice (Prins et al. 2001). In the present study we evaluated the effects of neonatal DES exposure on the SV of αERKO and βERKO mice.

## Materials and Methods

*Animals and treatment.* All studies involving animals were conducted in accordance with the National Institutes of Health *Guide for the Care and Use of Laboratory Animals* (Institute of Laboratory Animal Research 2011) and approved by the National Institute of Environmental Health Sciences (NIEHS) Animal Care and Use Committee. Animals were treated humanely and with regard for alleviation of suffering. The generation of ERα- and ERβ-null mice is previously described (Krege et al. 1998; Lubahn et al. 1993). Mice were generated by breeding C57/BL6 mice heterozygous for disruption of the ERα gene (*Esr1*) or the ERβ gene (*Esr2*) to produce homozygous ERα-null (αERKO) or ERβ-null (βERKO) mice, respectively, and WT littermates.

Pregnant females were housed under controlled lighting (12-hr light/dark cycle) and temperature conditions and were provided with NIH 31 laboratory mouse chow (Zeigler Brothers Inc., Gardners, PA) and fresh water *ad libitum*. On the day of parturition, considered day l of age, male offspring were pooled from multiple litters and randomly distributed among CD-1 foster mothers at eight per female. All offspring then received a subcutaneous injection of 2 μg (1–2 mg/kg/day) DES in corn oil or an equal volume of corn oil alone (vehicle) daily on PNDs 1–5. All offspring were weaned at 21 days of age and genotyped by polymerase chain reaction (PCR) on DNA extracted from tail biopsy using previously described methods (Couse et al. 2003). Mice in group 1 were killed at 10 weeks of age. Mice in group 2 were castrated at 10 weeks of age and allowed to recover for 10 days. Mice then received a subdermal implant of a 1-cm length of sealed Silastic tubing (1-cm in length, 1.47 mm inner diameter, 1.95 mm outer diameter) packed with crystalline dihydrotestosterone (DHT) or nothing (placebo) (implants were kindly provided by D. Handelsman, Sydney, Australia), and then were killed 2 weeks later (Lim et al. 2008). At necropsy, we recorded body weights, collected and heparinized whole blood from the inferior vena cava, and stored the plasma at –70°C until assayed. We collected the SV, trimmed off the coagulating gland, and recorded the wet weight. SVs were snap-frozen for RNA and protein analysis or fixed in paraformaldehyde solution for histological analysis. SV tissue sections (4 µm) were stained with hematoxylin and eosin.

*Hormone serum assays.* We evaluated serum estradiol and testosterone levels using Coat-A-Count radioimmunoassay kits (Siemens Healthcare Diagnostics, Los Angeles, CA) and were assayed using an APEX automatic gamma counter (ICN Micromedic Systems Inc., Huntsville, AL).

*RNA and protein isolation.* Frozen SV tissue from individual animals was pulverized, and the material was subdivided for either protein or RNA extraction. Total RNA was isolated using the RNeasy isolation kit (Qiagen Inc., Valencia, CA) according to the manufacturer’s protocol. Cytoplasmic and nuclear protein was extracted from frozen pulverized SV tissue using the NE-PER Protein Extraction Kit (Pierce, Rockford, IL) according to the manufacturer’s protocol.

*Reverse-transcriptase polymerase chain reaction (RT-PCR).* SV RNA (0.5 μg) was reverse transcribed using the SuperScript First Strand Synthesis Kit (Invitrogen, Carlsbad, CA) according to manufacturer’s protocol. As a negative control, we used a sample containing RNA but no reverse transcriptase. Real-time RT-PCR was performed using the ABI PRISM 7900HT Sequence Detection System (Applied Biosystems, Foster City, CA) and SYBR Green (Invitrogen). Primers were designed using Applied Biosystems Primer Express Software (version 2.0).

Real-time RT-PCR was performed using 2.5 ng cDNA. Samples were analyzed in duplicate, and a negative control sample was included on each plate. For all samples, the cyclophilin gene [peptidylprolyl isomerase A (*Ppia*)] was used as an endogenous control for normalization. Expression ratios were calculated using the mathematical model described by Pfaffl (2001):

2^–(Ct_gene of interest_ – Ct^*^_Ppia_^*^)^ × 10,000,

where Ct is cycle threshold.

*Western immunoblot analysis.* Cytoplasmic SV protein (1 µg) was immobilized to nitrocellulose membrane using the BioRad Dot Blot apparatus (Bio-Rad, Hercules, CA) according to the manufacturer’s protocol. We performed the protein analysis using a single blot that was stripped and reprobed. Equal loading was determined using MEMCode total protein stain (Pierce) and then destained before Western blotting. Nonspecific peroxidases were eliminated using 3% hydrogen peroxide, and nonspecific sites were blocked with 10% bovine serum albumin in Tris-buffered saline, pH 7.4, plus 0.1% Tween-20 (TBS-T). Blots were then incubated with primary antibodies for 1 hr at room temperature. Anti-mouse SV secretory protein IV (SVS IV) rabbit polyclonal antibody, a gift from T. Teng (NIEHS), and rabbit anti-mouse LF polyclonal antibody, isolated as described previously (Jefferson et al. 1996), were used at 1:5,000 dilution. Blots were then incubated with secondary antibody, anti-rabbit horseradish peroxidase (Amersham, Piscataway, NJ), diluted 1:10,000 in TBS-T. Membranes were washed five times for 5 min each in TBS-T, and immunoreactive bands were visualized using WestDura Reagent (Pierce) following the manufacturer’s instructions. Blots were exposed to film for 1 min, and images were captured by a camera (model c8484-54-03G) using LabWorks s46 software (both from UVP BioImaging Systems, Upland, CA).

*Statistical analysis.* The data were analyzed using JMP software (version 7) and SAS software (version 9.1), both from SAS Institute Inc. (Cary, NC). For body weight and SV wet weight, parametric tests were used to compare values. We evaluated real-time RT-PCR data using analysis of variance (ANOVA) followed by Tukey’s test. For estrogen and testosterone levels, statistical significance was determined using *t*-test, and groups were compared using nonparametric Mann-Whitney tests. *p*-Values < 0.05 were considered statistically significant.

## Results

**Figure 1:**
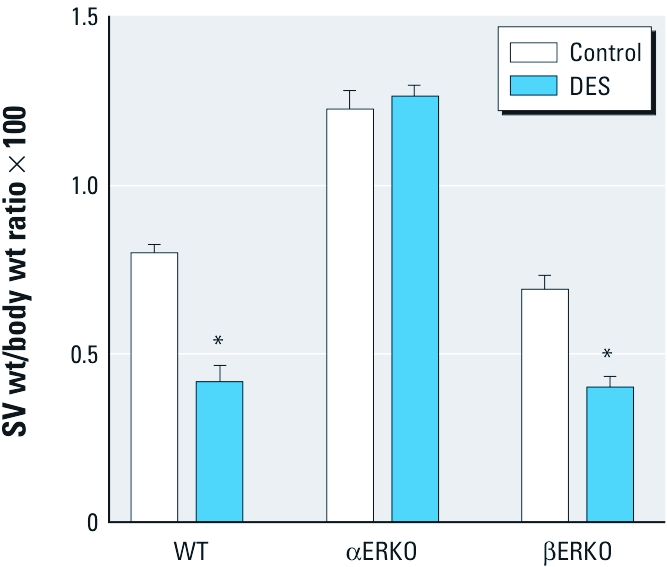
SV weight (wt) measured at 10 weeks of age in intact male WT, αERKO, and βERKO mice treated neonatally with vehicle (control) or DES (2 μg/day on days 1–5). Treatment groups: WT control, *n* = 19; WT DES, *n* = 18; αERKO control, *n* = 11; αERKO DES, *n* = 20; βERKO control, *n* = 14; βERKO DES, *n* = 20). Data are mean ± SE. *p < 0.05 compared with the corresponding control, by parametric test.

*DES Exposed* α*ERKO male mice retain androgen responsiveness in SV.* The SVs of adult WT males exposed to DES as neonates exhibited a 50% reduction in weight relative to untreated WT controls (Figure 1). This is consistent with previous reports (Prins et al. 2001) and indicates that DES treatments were successful. DES-exposed βERKO males exhibited a remarkably similar effect (Figure 1). In contrast, DES exposure had no measurable effect on SV weights in αERKO males. Instead, both DES-treated and control αERKO males exhibited significantly larger SV weights compared with age-matched WT and βERKO males (*p* < 0.05).

**Figure 2:**
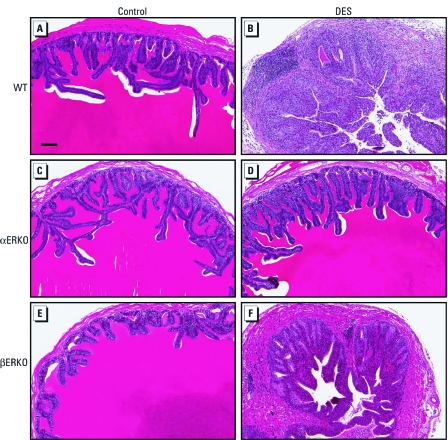
Pathology of SVs of male WT (*A,B*), αERKO (*C,D*), and βERKO (*E,F*) mice treated neonatally with vehicle (control; *A,C,E*) or DES (2 μg/day on days 1–5; *B,D,F*). SVs are stained with hematoxylin and eosin. Bar = 100 µm.

Neonatal DES exposure resulted in significant histological alterations in the SV of WT and βERKO males but not αERKO males (Figure 2). The SV of WT mice neonatally exposed to DES showed a significant increase in thickness of the smooth muscle layer, as well as the connective tissue that separates the epithelium from the smooth muscle layer (Figure 2B). The single columnar epithelial structure is no longer present, replaced by increased glandular formation. However, these glands do not appear to be secretory in nature because they did not produce the secretions seen in the control SV (Figure 2B). In addition, significant vasculature infiltration can be seen in the connective tissue layer, as well lymphocyte infiltration in the smooth muscle layer. DES-treated βERKO mice exhibited significant thickening and lymphocyte infiltration of the smooth muscle layer, similar to the histopathological effects observed in the WT mice. Despite potential hyperplasia of the epithelium, apparently some of the epithelium remained functional because epithelial cells still maintained a polarized columnar shape and produced some secretions (Figure 2F). In contrast, the well-documented histological identifiers of DES exposure in the SV exhibited by WT and βERKO males was not evident in the DES-treated αERKO males (Figure 2D).

**Figure 3:**
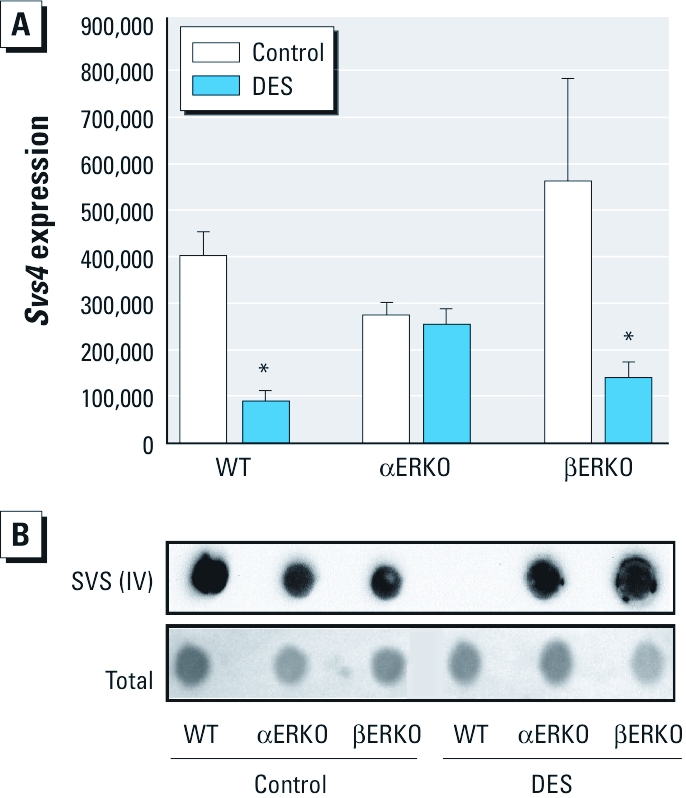
SVS IV expression in SVs of adult intact male mice (all three genotypes) treated neonatally on days 1–5 with vehicle (control) or 2 μg/day DES. Treatment groups: WT control, *n* = 12; WT DES, *n* = 12; αERKO control, *n* = 7; αERKO DES, *n* = 15); βERKO control, *n* = 8; βERKO DES, *n* = 15). (*A*) *Svs4 *expression in the SV by real-time RT-PCR; expression levels were normalized to the *Ppia *housekeeping gene. (*B*) SVS IV protein expression in SVs; Equal protein loading was confirmed with MEMCode total protein stain before immunoblotting. *p < 0.05 compared with the corresponding control, by ANOVA followed by Tukey’s test.

The expression of *Svs4*, a gene positively regulated by androgen in the mouse SV, was greatly reduced in the SVs of DES-exposed WT males (Figure 3A). This reduced *Svs4* expression was further confirmed by the absence of detectable SVS IV protein as evaluated by Western blot (Figure 3B). We observed a similar effect on *Svs4* expression in DES-exposed βERKO males, although levels of SVS IV protein were more variable in this genotype. Interestingly, βERKO mice retained SVS IV protein expression even though they had lower mRNA levels after DES exposure, unlike WT controls. These data suggest that some aspects of androgen response, such as maintenance of SVS IV protein, may require the action of ERβ and warrant further studies to evaluate a potential role for ERβ. In contrast, the SVs of DES-exposed αERKO males exhibited normal *Svs4* mRNA expression and SVS IV protein levels compared with controls.

Because maintenance of normal weights and *Svs4* expression in mouse SV are dependent on sufficient androgen stimulation, abnormally low SV weights and absence of *Svs4* expression and SVS IV protein observed in DES-exposed adult WT and βERKO males suggest that estrogen leads to a permanent disruption of androgen signaling in this tissue. Because the actions of testosterone and its potent metabolite DHT in target cells are dependent on the presence of the androgen receptor (AR), we assessed *Ar* in the SVs of the different genotypes and treatment groups by RT-PCR. Despite exhibiting what appears to be androgen resistance, SV tissues of DES-exposed WT and βERKO males exhibited normal levels of *Ar* when assessed by RT-PCR [see Supplemental Material, Figure 1 (http://dx.doi.org/10.1289/ehp.1103678)]. AR levels in the SV tissues of DES-exposed αERKO males were also comparable to WT levels. All genotypes exhibited a normal pattern of AR expression, regardless of neonatal treatment, when assessed by immunohistochemistry (see Supplemental Material, Figure 1).

Given that AR expression patterns were normal, we turned our focus to the possibility that DES exposure led to a reduction in circulating testosterone levels and hence reduced androgen signaling in the SV (Table 1). Surprisingly, we found that testosterone levels in DES-exposed WT males were somewhat elevated, although not statistically significant, compared with controls. In βERKO males, testosterone levels were normal in both treatment groups. Consistent with previous reports (Lindzey et al. 1998), untreated αERKO males exhibited significantly higher levels of circulating testosterone relative to WT and βERKO males (Table 1). Although neonatal DES exposure appeared to reduce testosterone levels in αERKO males, this difference was not significant compared with untreated ERα-null animals, and levels still remained 4- to 5-fold higher than those of WT males. Estradiol levels were comparable among all genotypes and treatment groups [see Supplemental Material, Table 1 (http://dx.doi.org/10.1289/ehp.1103678)].

**Table 1:** Serum levels in intact mice by genotype and treatment.

Control			DES		
Group	*n*	Mean ± SE	*n*	Mean ± SE	*p*-Value
Testosterone (ng/mL)										
WT		14		160.75 ± 91.22		16		220.77 ± 57.34		0.053
αERKO		11		1011.14 ± 107.24		20		739.23 ± 115.20		0.070
βERKO		6		257.38 ± 183.74		19		229.19 ± 96.56		0.510
Estradiol (pg/mL)										
WT		9		25.78 ± 3.58		12		28.46 ± 2.07		0.750
αERKO		6		23.34 ± 6.74		14		30.42 ± 3.08		0.360
βERKO		3		30.42 ± 3.54		15		26.71 ± 1.87		0.380
Statistical significance was determined between control and DES-treated groups within a given genotype by Mann-Whitney tests.										

**Figure 4:**
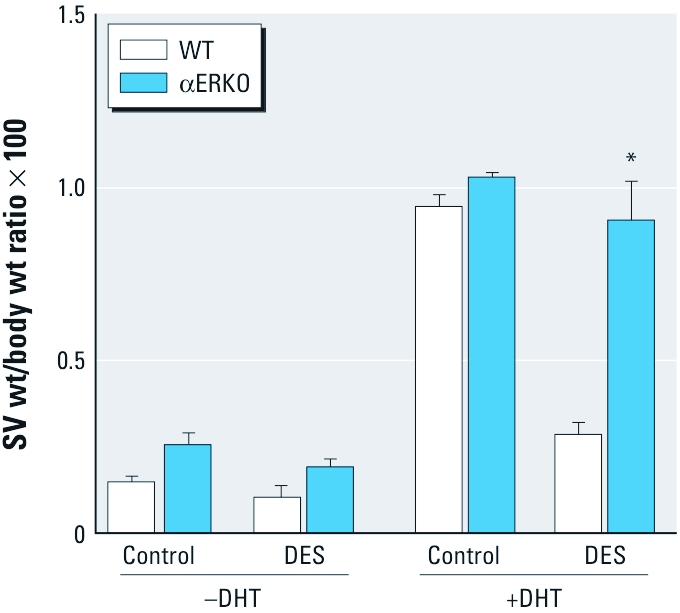
SV weight (wt) of male mice (WT and αERKO) treated neonatally on days 1–5 with vehicle (control) or 2 μg/day DES, castrated at 10 weeks of age, and exposed to DHT in Silastic tubing or Silastic tubing alone for an additional 2 weeks. Treatment groups: without (–) DHT: WT control, *n* = 11; WT DES, *n* = 6; αERKO control, *n* = 4; αERKO DES, *n* = 10; with (+) DHT: WT control + DHT, *n* = 8; WT DES + DHT, *n* = 6; αERKO control + DHT, *n* = 7; αERKO DES + DHT, *n* = 8). *p < 0.05 compared with the corresponding control, by parametric tests.

The above data indicate that neonatal DES exposure causes the SV tissues to become refractory to androgen stimulation during adulthood, despite exhibiting normal *Ar* expression patterns in a milieu of normal circulating testosterone levels. Furthermore, the absence of this effect in DES-exposed αERKO males strongly suggests that this effect is ERα-mediated during development. It is conceivable, however, that the remarkably high testosterone levels in αERKO mice (Table 1) may mask the effects of DES-induced androgen resistance, such as those observed in DES-exposed WT males. To test this hypothesis, control and DES-exposed WT and αERKO males were castrated at 10 weeks of age, provided a Silastic implant of placebo or DHT 10 days postsurgery, and killed 2 weeks later. In this milieu, we assumed that circulating androgen levels among the genotypes would now be comparable, allowing for more definitive evaluation of the role of ERα and the effects of neonatal DES exposure on the SV. As expected, castration led to an acute loss in SV weight in both WT and αERKO males regardless of neonatal treatment (Figure 4). Two weeks of DHT treatment totally restored SV weights in castrated control WT males but had no effect in DES-exposed WT males. These data definitively demonstrate that developmental exposure to DES leads to androgen resistance in the adult SV. In contrast, DHT treatment fully restored castration-induced losses in SV weights in both control and DES-exposed αERKO males. These data indicate that αERKO males are largely resistant to the effect of neonatal DES exposure on the SV, providing convincing evidence of the importance of ERα in mediating the toxicological effects of DES.

**Figure 5:**
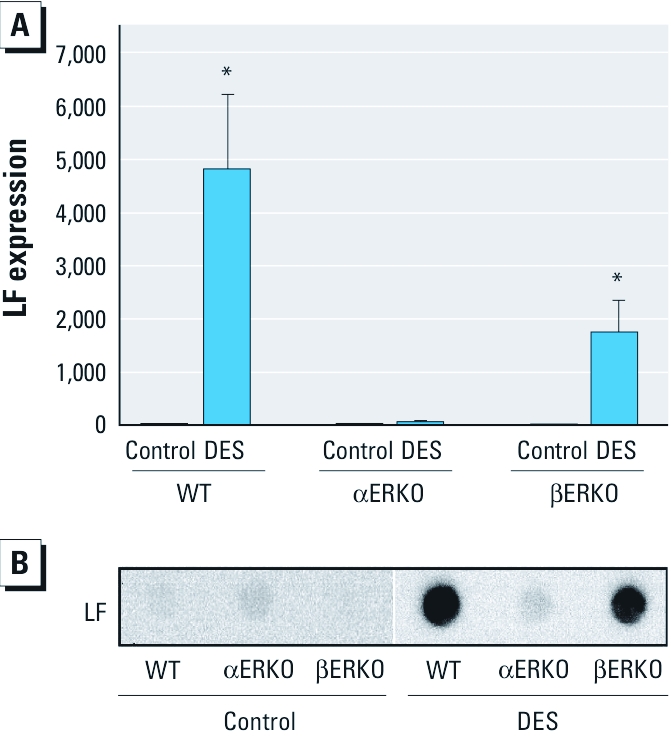
LF expression in SVs of adult intact male mice (all three genotypes) treated neonatally on days 1–5 with vehicle (control) or 2 μg/day DES. Treatment groups: WT control, *n* = 12; WT DES, *n* = 12; αERKO vehicle, *n* = 7; αERKO DES, *n* = 15; βERKO control, *n* = 8; βERKO DES, *n* = 15. (*A*) *Ltf *expression in the SV by real-time RT-PCR. All expression levels were normalized to the *Ppia *housekeeping gene. (*B*) LF protein expression in SVs; equal protein loading was confirmed with MEMCode total protein stain before immunoblotting (data not shown). *p< 0.05 compared with the corresponding control, by ANOVA followed by Tukey’s test.

*ER*α *mediates DES-induced molecular feminization of SV.* The LF gene (*Ltf*) is an estrogen-responsive gene in the uterus and that is not normally expressed at measurable levels in the SV. However, previous studies have shown ectopic LF expression in the SV after neonatal DES exposure in mice (Beckman et al. 1994; Newbold et al. 1989), indicating DES-induced “feminization” of the tissue. As we expected, in the present study, *Ltf* expression and LF protein were undetectable in SVs of control adult males in the thre genotypes (Figure 5). In contrast, *Ltf* expression and protein were easily detected in the SVs of DES-exposed WT males. We also observed the same effect in DES-exposed βERKO males, whereas *Ltf* expression in the SVs of αERKO males remained absent regardless of neonatal treatment, indicating that DES-induced feminization is ERα mediated.

## Discussion

Our findings in this study demonstrate the importance of ERα in mediating the toxicological effects of neonatal DES exposure in the male SV. In WT and βERKO males, DES exposure resulted in a reduction in SV weight, ectopic expression of *Ltf* mRNA with a concurrent decrease in *Svs4* mRNA expression, and a permanent resistance to androgenic stimulation that was not a result of decreased AR levels; these findings suggest an ERα-mediated down-stream interference with androgen action as a function of early-life estrogenic exposure. The detrimental and permanent effects of developmental exposure to DES in the mouse male reproductive tract have been relatively well described (Marselos and Tomatis 1993; Prins 1992; Prins et al. 2001). In the present study, the hallmark histological alterations of DES exposure were evident in WT mice. However, the penetrance of these effects varied in the βERKO males, whereas αERKO males were resistant to the detrimental histological alterations. Existing evidence that the toxicological effects of developmental exposure to DES in the female reproductive tract and the prostate of males are dependent on functional ERα at the time of exposure is quite convincing (Couse et al. 2001; Couse and Korach 2004; Prins et al. 2001). Because many xenoestrogens, including DES, possess properties that can potentially disrupt cellular functions apart from their ability to act as estrogens, research toward a thorough understanding of their toxicology has been problematic. Results of investigations such as the present study using receptor-null mice add to the growing evidence that the detrimental effects of DES are dependent on functional ERα and therefore are attributable to its hormonal properties and ability to disrupt endogenous estrogen signaling. These data definitively show that the developing male reproductive tract, although perhaps not dependent on estrogens or ER for normal development, expresses ER and is quite sensitive to the toxicological effects of exogenous estrogens when aberrantly exposed. Although the risk of DES exposure among humans, especially during development, has long been diminished (since the mid-1970s), the toxicological effects of developmental exposure to DES are largely accepted as a harbinger of similar potentials among environmental and industrial estrogenic compounds, such as genistein and bisphenol A, to which humans are thought to be exposed.

Perhaps the most prominent effect of developmental DES exposure in the reproductive tract of male mice is a permanent inability of the SVs to reach and maintain their normal size during adulthood. This hallmark effect of DES appears to be due to induction of a permanent resistance to androgen stimulation, on which growth of the SVs is so dependent, because it could not be rescued by a castration/hormone replacement scheme where animals were provided pharmacological levels of androgen for 2 weeks. Further evidence of androgen resistance in the SVs after DES exposure is the absence of androgen-induced *Svs4* expression during adulthood. Somewhat similar morphological and biochemical indicators of androgen resistance are observed in the prostate of DES-exposed mice. However, unlike in the prostate where this effect of DES appears to be due at least in part to a permanent reduction in AR expression (Prins 1992; Prins et al. 2001), we demonstrate here that DES-induced androgen resistance in the SVs occurs in the presence of normal AR expression patterns and circulating testosterone levels. Our findings are in agreement with other reports showing AR levels are not significantly reduced in neonatally exposed SV (Turner et al. 1989). These data suggest that the androgen signaling pathway is vulnerable to developmental exposure to DES at multiple points, both at the level of the AR, as in the prostate, and at points presumably downstream of the AR, as appears to be the case in the SV. This latter mechanism may involve altered expression of a coregulator that is critical to AR function.

The difference in apparent mechanisms by which DES exerts its effects in the prostate versus SV may be due to the tissues originating from a different embryonic anlagen, or the developmental stage of each at the time of exposure. The epithelium of the prostate is from the endodermal urogenital sinus, and the epithelium of the SV is from the Wolffian duct. The expression of AR is developmentally regulated, and expression occurs in different parts of the male reproductive tract temporally, suggesting some differences in developmental programming (Cooke et al. 1991).

In addition to permanent androgen resistance in the SV brought about by neonatal DES exposure, feminized patterns of gene expression also occur. *Ltf* expression is largely limited to the epithelial tissues of the female reproductive tract under stringent regulation by estradiol/ERα actions and is not normally expressed in the male reproductive tract. Previous studies, however, have shown that mice exposed prenatally to DES express significantly high levels of *Ltf* in the SV (Beckman et al. 1994; Newbold et al. 1989; Pentecost et al. 1988). Here we demonstrate that this DES-induced feminization does not occur in the absence of ERα but remains in the absence of ERβ. These data demonstrate that ERα is fundamental to the molecular feminization of the SV. It remains unclear whether the observed feminization is also due to the onset of androgen resistance, leading to an environment of unopposed estradiol stimulation, or is attributable to a wholly separate disruption in normal signaling. Studies have shown that uteri of female mice exposed developmentally to DES also have aberrantly high expression of *Ltf* in the absence of estrogen (Nelson et al. 1994; Newbold et al. 2007). This dysregulation has been associated with patterns of hypomethylation of CpG sites near the estrogen response element in the *Ltf* gene in uterine tissues of DES-exposed mice (Li et al. 1997). The ectopic expression of *Ltf* in the SV is plausibly due to a similar mechanism, and the absence of this ectopic expression in mice lacking ERα suggests that this receptor is involved in the epigenetic changes associated with neonatal DES exposure. Further studies investigating epigenetic changes using αERKO mice will help elucidate molecular events that lead to permanent alterations in gene expression.

## Conclusion

The data presented here demonstrate that ERα plays a role in the developmental effects resulting from DES toxicity in the SV. ERα is involved in the lack of androgen responsiveness determined by increases in SV weight after DHT treatment and by SVS IV protein and *Svs4* gene expression, but this does not appear to be due to down-regulation of the AR itself. Therefore, other factors that control androgen signaling must be affected. In addition, this study definitively shows that ERα is necessary for the molecular feminization of the SV after neonatal exposure to DES, because we did not observe aberrant LF expression in αERKO mice.

Irrespective of the underlying mechanisms, the toxicological effects of DES that lead to androgen resistance and feminization in the SV are dependent on functional ERα. Furthermore, this is clearly a toxicological effect of aberrant stimulation of ERα signaling in the SV during development, as unexposed αERKO males invariably exhibited overly well-maintained SVs, thus indicating that normal development and function of the tissue are not dependent on functional ERα.

## Supplemental Material

(352 KB) PDF
