# Supplementary material for: Estrogen Receptor-α Mediates Diethylstilbestrol-Induced Feminization of the Seminal Vesicle in Male Mice
Source: Environ Health Perspect. 2012 Jan 24;120(4):560–5. doi: 10.1289/ehp.1103678 (PMC3339448; doi:10.1289/ehp.1103678)
Supplement: (352 KB) PDF [file ehp.1103678.s001.pdf]

Estrogen Receptor Alpha (ERα) Mediates Diethylstilbestrol (DES)-induced Feminization of the Seminal Vesicle in Male Mice

Vickie R. Walker[1], Wendy N. Jefferson[2], John F. Couse[1,3] and Kenneth S. Korach[1]

[1] Receptor Biology Section, Laboratory of Reproductive and Developmental Toxicology, National Institutes of Environmental Health Sciences, National Institutes of Health, Research Triangle Park, North Carolina 27709
[2] Reproductive Medicine Group, Laboratory of Reproductive and Developmental Toxicology, National Institutes of Environmental Health Sciences, National Institutes of Health, Research Triangle Park, North Carolina 27709
[3] Taconic Inc., Germantown, NY 12526

“Supplemental Material”

Table of Contents

Figure 1 2

Table 1. 3

Figure 2 4

AR Immunohistochemistry Materials and Methods...5

Figure 1

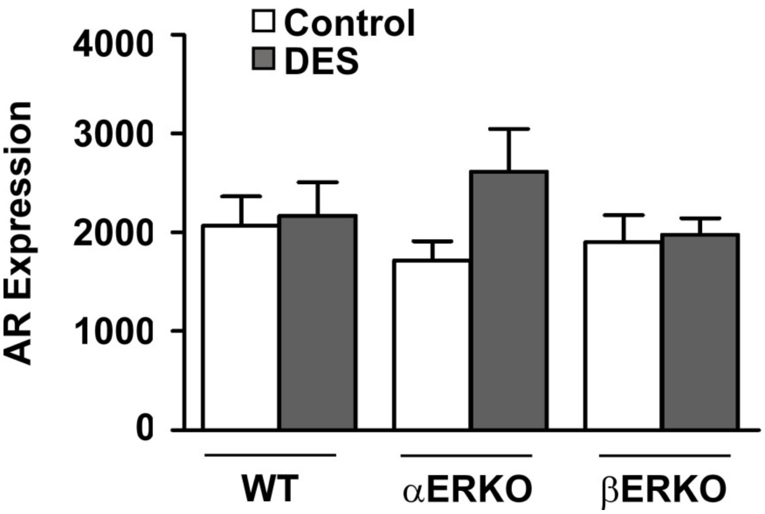

Figure 1: Androgren receptor expression by real time RT-PCR in seminal vesicles of adult intact male mice treated neonatally with vehicle or DES (2 ug/pup/day on days 1-5)in WT(vehicle n=12 and DES n=12), αERKO(vehicle n=7 and DES n=15) and βERKO (vehicle n=8 and DES n=15) mice. All levels were normalized to cyclophilin as a housekeeping gene. There is no significant reduction in AR mRNA expression among treatment groups or across genotypes. The AR levels in all three genotypes were unaffected by neonatal DES exposure. Statistical significance was determined using analysis of variance (ANOVA) followed by Tukey's test. * P-values less than 0.05 were considered statistically significant.

Table 1.

Statistical Evaluation of Serum by Genotype

Group	Controls	DES
Testosterone	p-value	p-value
Wild Type vs αERKO	0.001	0.002
Wild Type vs βERKO	0.17	0.28
αERKOvs βERKO	0.017	0.0005

Group	Controls	DES
Estradiol p-values	p-value	p-value
Wild Type vs αERKO	0.60	0.87
Wild Type vs βERKO	0.72	0.63
αERKOvs βERKO	0.71	0.43

Serum levels of testosterone and estradiol were measured in intact males (same samples as Table 1). Two sample t-test were performed to test for significance between genotypes. In both control and DES treated males, testosterone levels differed significantly between wild type and αERKO and between βERKO and αERKO, but not between wild type and βERKO. There were no significant differences in estradiol levels between genotypes.

Figure 2

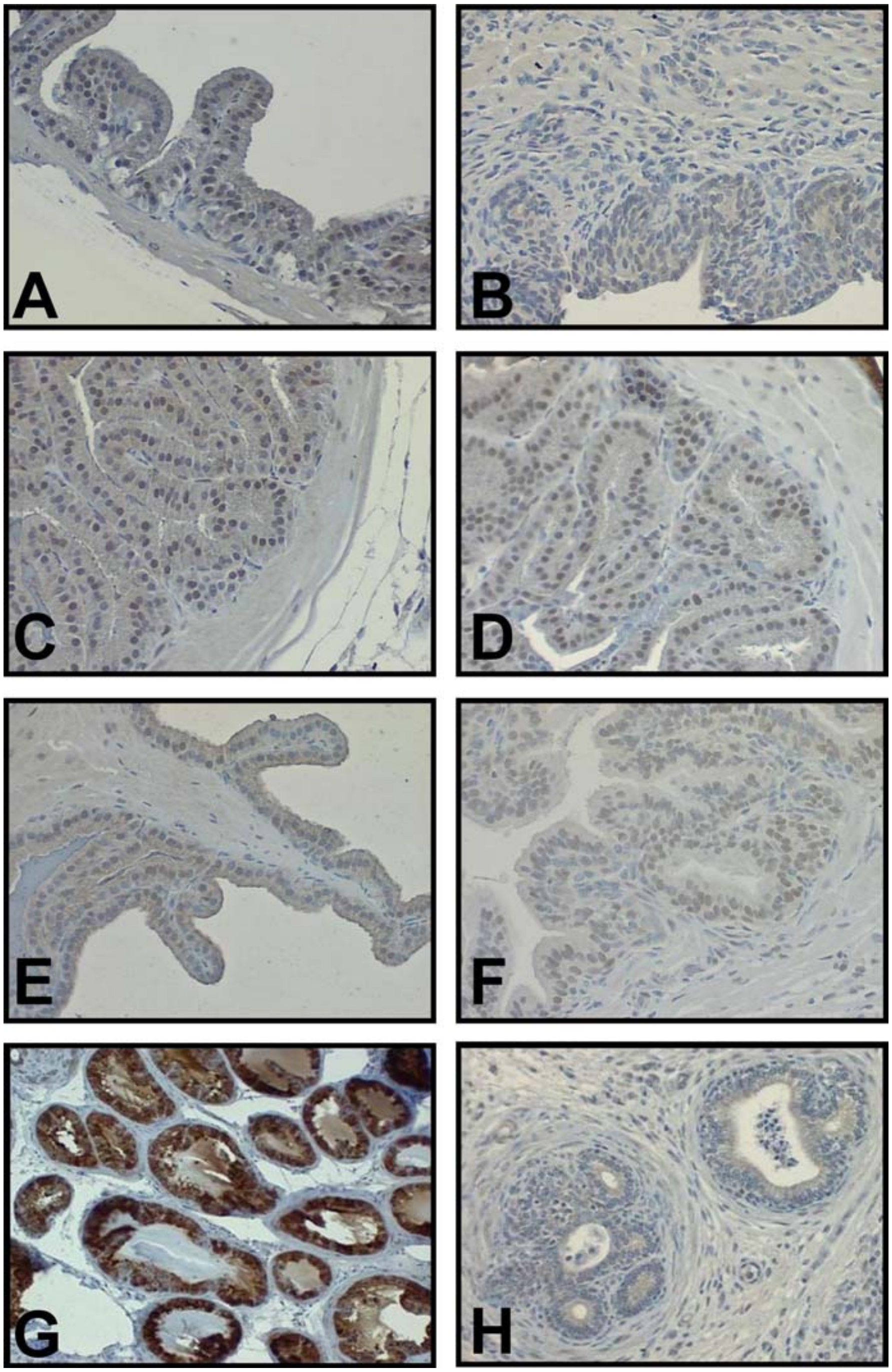

Figure 2: Immunohistochemistry of androgen receptor (AR) levels in male reproductive tract tissues. Panel A: WT vehicle control seminal vesicle. Panel B: WT DES seminal vesicle. Panel C: αERKO vehicle control seminal vesicle.

Panel D: αERKO DES seminal vesicle. Panel E: βERKO vehicle control seminal vesicle. Panel F: βERKO DES seminal vesicle. Note low level AR expression in the seminal vesicle epithelium of all treatment groups and genotypes. Panel G: WT control ventral prostate (positive control). Panel H: WT DES ventral prostate. Note lower level of expression than WT control. A minimum of 3 individual mice from each treatment group was assessed for AR expression in the seminal vesicle and a representative from each group in shown in this figure. All photomicrographs are 200X magnification.

AR Immunohistochemistry Materials and Methods

Male reproductive tract tissues were collected and fixed in cold 10% neutral buffered formalin for 24 hours. Tissues were then processed for histology, embedded in paraffin and cut at 6 microns. Sections were deparaffinized in a graded series of ethanol and then endogenous peroxidases were blocked with 3% hydrogen peroxide for 10 min. Antigen retrieval was performed using Citrate buffer (Biocare Medical, Concord, CA) in a decloaker (Biocare Medical) for 5 min. Sections were rinsed with TBS-T and non-specific sites were blocked with 10% BSA for 30 min. Sections were then incubated with antibody to AR (1:50, Upstate Biotechnology, Lake Placid, NY) for 1 hour followed by washes with TBS-T. Biotinylated anti-rabbit (Vector Laboratories, Burlingame, CA) was incubated on the sections for 30 min followed by ExtrAvidin peroxidase (Sigma Chemical Company, St. Louis, MO). Immunoreactive product was visualized with DAB(3,3'-diaminobenzidine) and counterstained with hematoxylin.
